# Body image change and improved eating self-regulation in a weight management intervention in women

**DOI:** 10.1186/1479-5868-8-75

**Published:** 2011-07-18

**Authors:** Eliana V Carraça, Marlene N Silva, David Markland, Paulo N Vieira, Cláudia S Minderico, Luís B Sardinha, Pedro J Teixeira

**Affiliations:** 1 Faculty of Human Kinetics, Technical University of Lisbon, Estrada da Costa, 1495-688, Cruz Quebrada, Portugal; 2 School of Sport, Health and Exercise Sciences, Bangor University, George Building, Holyhead road, Bangor, Gwynedd, UK

**Keywords:** Body image, Eating Self-regulation, Eating behavior, Weight Management, Obesity

## Abstract

**Background:**

Successful weight management involves the regulation of eating behavior. However, the specific mechanisms underlying its successful regulation remain unclear. This study examined one potential mechanism by testing a model in which improved body image mediated the effects of obesity treatment on eating self-regulation. Further, this study explored the role of different body image components.

**Methods:**

Participants were 239 overweight women (age: 37.6 ± 7.1 yr; BMI: 31.5 ± 4.1 kg/m^2^) engaged in a 12-month behavioral weight management program, which included a body image module. Self-reported measures were used to assess evaluative and investment body image, and eating behavior. Measurements occurred at baseline and at 12 months. Baseline-residualized scores were calculated to report change in the dependent variables. The model was tested using partial least squares analysis.

**Results:**

The model explained 18-44% of the variance in the dependent variables. Treatment significantly improved both body image components, particularly by decreasing its investment component (*f^2 ^*= .32 vs. *f^2 ^*= .22). Eating behavior was positively predicted by investment body image change (p < .001) and to a lesser extent by evaluative body image (p < .05). Treatment had significant effects on 12-month eating behavior change, which were fully mediated by investment and partially mediated by evaluative body image (effect ratios: .68 and .22, respectively).

**Conclusions:**

Results suggest that improving body image, particularly by reducing its salience in one's personal life, might play a role in enhancing eating self-regulation during weight control. Accordingly, future weight loss interventions could benefit from proactively addressing body image-related issues as part of their protocols.

## Background

Overweight and obesity remain highly prevalent in Western cultures and constitute a major cause of preventable co-morbidities and death [1-3]. Further, they are associated with substantial health care costs [3]. The treatment of obesity is problematic and weight loss interventions generally result in modest effects [4]. Improving intervention efficacy remains a critical challenge and identifying mechanisms or factors (i.e., mediators) which facilitate adherence to health-related behaviors critical to successful weight management, such as healthy eating and exercise behaviors, will contribute to more successful interventions in the future.

Since obesity is a product of energy imbalance and thus highly reliant on dietary energy intake and energy expenditure, it is not surprising that healthy weight management almost always involves the successful regulation of eating behavior. Several studies indicate that eating-related behaviors such as high flexible restraint, high eating self-efficacy, reduced disinhibition and emotional eating, and low hunger predict positive outcomes in obesity treatment [5-7]. At the same time, body image problems are highly prevalent in overweight and obese people [8] especially among those seeking treatment [e.g., [9]] and can undermine successful weight management, predicting poorer weight outcomes and increasing chances of relapse [6,8,10,11]. A relatively large body of evidence indicates that there are associations between a range of body image disturbances and problematic eating behaviors and attitudes [c.f., [12-14]]. Therefore, improving body image might be a potential mechanism involved in the successful regulation of eating behaviors and obesity treatment is a critical setting to test this hypothesis.

Not only is there evidence that body image experiences predict the severity of problematic eating patterns, but longitudinal and structural modeling investigations also point to poor body image as a precursor of the adoption of dysfunctional eating behaviors among other unhealthy weight control strategies [e.g., [15-18]]. For instance, Neumark-Sztainer and colleagues (2006) showed that lower levels of body satisfaction were associated with more health-compromising behaviors, such as unhealthy weight control behaviors and binge eating, five years later [18]. Further, sociocultural models of bulimia nervosa assign body image concerns a causal role in the development of disordered eating [17]. Stice proposed that sociocultural pressures to be thin, widespread in Western cultures, lead women to internalize a slender body as the standard for feminine beauty [19]. Consequently, this internalization can result in the experience of a discrepancy between the ideal and one's actual figure and prompts body dissatisfaction and over-concern, since the ideal body weight is often very low and thus achievable by only a few. Weight/body dissatisfaction, in turn, could motivate extreme and unhealthy behaviors in an effort to lose weight, which in turn might increase the risk of developing binge eating and other disturbed eating behaviors [17,19]. These findings have led researchers to conclude that body image distress is one of the most potent risk factors for eating disturbances [20].

Body image comprises two attitudinal dimensions. Evaluative body image refers to cognitive appraisals and associated emotions about one's appearance, and it includes self-ideal discrepancies and body satisfaction-dissatisfaction valuations [21]. In contrast, body image investment refers to the cognitive-behavioral importance of appearance in one's personal life and its salience to one's sense of self. This dimension reflects a dysfunctional investment in appearance characterized by an excessive preoccupation and effort devoted to the management of appearance, as opposed to a more adaptive valuing and managing of one's appearance [21]. This structure of attitudinal body image has been empirically supported indicating that although the optimal prediction of poor/negative body image requires both evaluative and investment aspects of body image, the former is not sufficient per se to produce body image distress [22]. Similarly, both body image components were found to predict eating disturbance, although body image investment presented greater predictive power, in some cases surpassing the effects of evaluative body image [21,23]. For example, Cash, Phillips, et al. [23] found that body image investment had not only a greater but also a unique, independent contribution to the prediction of disturbed eating attitudes, above and beyond a simple index of body dissatisfaction.

As Bruch originally argued, amelioration of dysfunctional body image is often necessary for effectively treating and improving disturbed eating behaviors [24]. Obesity treatment seems to be effective in improving body image even with modest weight losses [e.g., [25,26]]. Thus, the purpose of the present study was to examine whether body image (positive) change during a weight loss intervention comprising a body image module would mediate the successful regulation of eating behavior by testing a three-level model in which treatment would enhance body image (evaluative and investment components), which in turn would improve the regulation of eating behavior. Further, this study analyzed whether the change in body image investment presented stronger effects on the regulation of eating behavior than evaluative body image.

## Methods

### Study Design and Intervention

This study was part of a randomized controlled trial including a 1-year behavior change intervention, primarily aiming at increasing physical activity and energy expenditure, adopting a moderately restricted diet, and ultimately establishing exercise and eating patterns consistent with sustainable weight loss/maintenance. Participants were randomly assigned to intervention and control groups. The comparison group received a general health education curriculum based on several educational courses on various topics (e.g., preventive nutrition, stress management, self-care, and effective communication skills). The intervention included 30 group sessions covering topics such as physical activity, emotional and external eating, improving body acceptance and body image, among other cognitive-behavioral aspects (e.g., identifying personal barriers, overcoming lapses, defining adequate goals, and implementing self-monitoring). The program's principles and style of intervention were based on self-determination theory [27,28] with a special focus on increasing competence and internal regulation toward exercise and weight control, while supporting participants' autonomous decisions as to which changes they wanted to implement and how.

Regarding body image enhancement, the intervention aimed at increasing participants' body acceptance and satisfaction and at decreasing their over-preoccupation and dysfunctional investment in appearance. For that purpose, several strategies were implemented within this intervention module. Some were predominantly used to improve evaluative body image while other strategies were essentially intended to reduce dysfunctional body image investment. Asking participants to view and gradually explore their body and its parts, in front of a mirror, in the privacy of their home; establishing more realistic goals and expectations for themselves and their weight/body, by confronting their ideal physique with the real limits in their biological capacities to meet their goals (e.g., observe their own and their parents weight history); and providing dance and relaxation classes were the main strategies employed to improve the evaluative component. To reduce dysfunctional investment in appearance, the following key strategies were implemented: helping participants understand the concept of body image (i.e., a subjective construct, independent of physical appearance) and recognize the social and personal roots of their own body image development; asking participants to keep a self-monitoring diary to record critical body image experiences in which they feel self-conscious, their beliefs in the situation (e.g., thoughts, self-statements, negative "body talk"), and the associated emotional and behavioral consequences; helping participants cope with stereotypes and prejudice, facilitating the abandonment of the idea that they must look different to be happier; and working on cognitive restructuring to help participants challenge their maladaptive assumptions about appearance and its salience to their life and self-worth, by promoting the evaluation of evidence for and against their beliefs and the construction of alternative thoughts. It should be noted that effectively isolating and specifically targeting one body image component (e.g. evaluative) without affecting another related component (e.g. investment) is a difficult task; they are dimensions of a higher-order construct and as such they will naturally covary.

A detailed description of the study's theoretical rationale, protocol, and intervention strategies can be found elsewhere [29,30]. The Ethics Committee of the Faculty of Human Kinetics - Technical University of Lisbon reviewed and approved the study.

### Participants

Participants were overweight or obese Portuguese women recruited from the community through web and media advertisements and announcement flyers to participate in a university-based behavioral weight management program. To be included, participants had to be women, between 25-50 years old, pre-menopausal, with a BMI between 25-40 kg/m^2^, be willing to attend weekly meetings (during 1 year), be free from major illnesses, and not taking medication known to interfere with weight regulation. Of all women who entered the study (N = 258), 19 women were subsequently excluded from all analyses because they started taking medication capable of affecting weight (n = 10), were diagnosed with serious chronic disease or severe illness/injury (n = 4), became pregnant (n = 2), or entered menopause (n = 3). These women were of similar age (p = .575) and BMI (p = .418) to the 239 considered as the effective initial sample. Of these, 201 completed assessments at the end of the intervention (12 months). T-tests comparing the complete dataset group (n = 170) vs. the missing dataset group (n = 31) were performed. No significant differences were found between the two groups for BMI, weight and height, which suggests data were missing completely at random (MCAR) and analyses would likely yield unbiased parameter estimates [31,32]. The mean age for the complete data group was 38.0 (SD 6.8 years) and the mean BMI was 31.3 (SD 4.0 kg/m^2^). All participants signed a written informed consent prior to participation in the study.

### Measures

#### Body Image

A comprehensive battery of psychometric instruments recommended in the literature was used to assess the two attitudinal components of body image, evaluative and investment [33]. To assess the evaluative component of body image, herein represented by self-ideal body discrepancy, the Figure Rating Scale (FRS) was used [34]. This scale comprises a set of 9 silhouettes of increasing body size, numbered from 1 (very thin) to 9 (very heavy), from which respondents are asked to indicate the figure they believed represented their current (i.e., perceived body size) and ideal body size. Self-ideal discrepancy was calculated by subtracting the score for ideal body size from the perceived body size score. Higher values indicate higher discrepancies.

The dysfunctional investment component was represented by body shape concerns and social physique anxiety. Body concerns were evaluated with the Body Shape Questionnaire (BSQ) [35,36], a 34-item instrument scored on a 6-point Likert-type scale (from 'never' to 'always'), developed to measure concern about body weight and shape, in particular the experience of "feeling fat" (e.g., "Has being naked, such as when taking a bath, made you feel fat?"), but also to measure several cognitive-behavioral consequences of those feelings (e.g., "Has thinking about your shape interfered with your ability to concentrate?", "Have you avoided wearing clothes that make you aware of your body?"). This instrument addresses the salience of body image in one's personal life, rather than merely asking about body image satisfaction [37], where higher values represent greater body shape concerns and greater salience. The Social Physique Anxiety Scale (SPAS) [38] was used to measure the degree to which people become anxious and concerned when others observe or evaluate their physiques, thereby assessing body image affective and cognitive features in a social environment. This scale comprises 12 items (e.g. ''Unattractive features of my physique make me nervous in certain social settings'') rated on a 5-point Likert-type scale (from 'not at all' to 'extremely'). Items 1, 5, 8, and 11 are reversed scored. Higher scores represent greater social physique anxiety. In evaluating the measurement model (see below) cross-loadings of items between these two scales (BSQ and SPAS) were analyzed, and items with cross-loadings above .60 were removed.

#### Eating Self-Regulation

Eating self-regulation (ESR) can be defined as the attempt to manage dietary intake in a mindful, voluntary and self-directed way (e.g., to achieve and maintain energy balance or weight loss), within the context of other physiological and environmental constraints [39]. In the current study, eating self-regulation referred to aspects known to positively influence weight management, namely high eating self-efficacy, high flexible cognitive restraint, reduced disinhibition (emotional, situational, and habitual), and reduced perceived hunger.

Eating self-efficacy was assessed with the Weight Efficacy Lifestyle Questionnaire (WEL) [40], by asking individuals to rate their confidence for successfully resisting opportunities to overeat and for self-regulating their dietary intake on a 10-point scale, ranging from "not confident at all" to "very confident". Higher scores represent greater eating self-efficacy. Cognitive restraint, disinhibition, and perceived hunger were measured with the 51-item Three-Factor Eating Questionnaire (TFEQ) [41]. Cognitive restraint reflects the conscious intent to monitor and regulate food intake (21 items). However, this global concept might include several behavioral strategies varying in their effectiveness in establishing a well self-regulated eating behavior. Hence, Westenhoefer noted the need to refine this concept and proposed its division into flexible and rigid types of restraint [42]. Rigid restraint (7 items) is defined as a dichotomous, all-or-nothing approach to eating and weight control, whereas flexible restraint (7 items) represents a more gradual approach to eating and weight control, for example, with "fattening" foods being eaten in limited quantities without feelings of guilt. Since flexible restraint is associated with low emotional and disinhibited eating, as opposed to rigid restraint, only the former subscale was considered in the present study as representing a better self-regulation of eating behavior. Higher scores indicate greater levels of flexible restraint. Disinhibition refers to an uncontrolled overconsumption of food in response to a variety of stimuli, such as situational and cognitive/emotional states (16 items). Taking into account the complexity of eating behavior, Bond and colleagues suggested the need for measuring and analyzing these factors at a more precise and domain-specific level [43]. Thus, disinhibition was also divided into three subscales: habitual, emotional, and situational susceptibility to disinhibition [43]. Habitual susceptibility (to disinhibition) describes circumstances that may predispose to recurrent disinhibition (e.g., "Do you go on eating binges though you are not hungry?"); emotional susceptibility is associated with negative affective states (e.g., "When I feel lonely, I console myself by eating"); and situational susceptibility which is fostered by specific environmental cues, such as social occasions (e.g., "I usually eat too much on social occasions"). This distinction allowed for higher item loadings and greater internal consistency of this construct. Perceived hunger refers to the extent to which respondents experience feelings and perceptions of hunger in their daily lives. Disinhibition and perceived hunger items were reverse scored, so that higher scores represented lower levels of these variables (and more positive eating self-regulation).

Assessments occurred at baseline and at 12 months. To report the change in body image and eating measures, baseline-residualized scores were calculated, where the 12-month variable is regressed onto the baseline variable [44]. Subjects completed the Portuguese versions of all questionnaires cited above. Forward and backward translations between English and Portuguese were performed for all the questionnaires. Next, two bilingual Portuguese researchers subsequently reviewed the translated Portuguese versions, and minor adjustments were made to improve grammar and readability. Cronbach's alphas for baseline and 12-month measurements were acceptable (above 0.70), except for flexible restraint which was slightly lower [5].

### Analytical Procedure

The theoretical model was tested using partial least squares (PLS) analysis with the SmartPLS Version 2.0 (M3) software [45]. PLS is a prediction-oriented structural equation modeling approach that estimates path models involving latent variables (LVs) indirectly measured by a block of observable indicators. Three reasons justify the use of PLS in this study. First, PLS is especially suitable for prediction purposes [46], since it explicitly estimates the latent variables as exact linear aggregates of their respective observed indicators. Second, PLS uses non-parametric procedures making no restrictive assumptions about the distributions of the data [47]. Third, unlike the covariance-based structural equation modeling approach (e.g., LISREL), PLS is appropriate for use with small sample sizes [48], due to the partial nature of the estimation procedure.

The PLS model was analyzed in two stages. In the first stage, the measurement model was tested. Item reliability was assessed by checking the loadings of the items on their respective latent variables. Items that were statistically significant and had loadings greater than .40 were retained [49]. The internal consistency of each scale was assessed by examining their composite reliability (CR). A CR of .70 or higher represents acceptable internal consistency [50]. Convergent and discriminant validity were assessed by examining the average variance extracted (AVE). Convergent validity exists when the latent variable explains on average 50% or more of the variance in its indicators, that is, when the AVE is at least .50 [50]. Discriminant validity is satisfied when the AVE for a latent variable is greater than its squared bivariate correlation with any other latent variable [50].

In the second stage, the structural model was tested. Three higher-order latent variables were defined. Investment BI was specified as a second-order variable with body shape concerns and social physique anxiety as its lower-order latent indicators; disinhibition was specified as a second-order variable with habitual, emotional, and situational susceptibility to disinhibition as its lower-order latent indicators; and eating self-regulation was specified as a third-order variable with flexible restraint, disinhibition, perceived hunger, and eating self-efficacy as its lower-order latent indicators. All latent variables were specified as reflective. The standardized path coefficients between latent variables (β) and the variance explained in the endogenous variables (*R*^2^) were examined. Structural paths were retained if they were statistically significant. Where there were significant intervening paths connecting distal variables, tests of mediation were conducted using the bootstrapping procedures incorporated in SmartPLS. When examining mediating effects, past work has shown the bootstrapping approach to be superior to the alternative methods of testing mediation, such as the Sobel test, with respect to power and Type I and II error rates [51]. Baron and Kenny's [52] formal steps for testing mediation were also followed. Full mediation is present when the indirect effect is significant, and there is a direct effect in the absence of the intervening variable (C path) that becomes non-significant in its presence (C' path). Partial mediation is present when the C' path is reduced but remains significant [53]. In addition, the ratio of the indirect effects to the direct effects was calculated to express the strength of the mediation effects [54].

As mentioned earlier, PLS does not make data distribution assumptions, thus parametric tests for the significance of the estimates are not available. Instead, SmartPLS employs a bootstrapping procedure to assess the significance of the parameter estimates. In the present analyses 5000 bootstrap samples with replacement were requested. SmartPLS does not provide significance tests for the *R*^2 ^values for dependent latent variables. Therefore, the effect sizes of the *R*^2 ^values (Cohen's *f *^2^) were calculated. Effect sizes of .02, .15, and .35 are considered small, medium, and large, respectively [44].

## Results

The central focus of this study was to test a three-level model by which a behavioral weight control intervention, encompassing a body image component, produced effects on eating self-regulation. The main effects of the intervention on weight and key psychosocial variables are described elsewhere [55]. In brief, at the end of the intervention (12 months), average weight loss was higher in the intervention group (-7.3 ± 5.9%) than in the control group (-1.7 ± 5.0%), and so was the percentage of participants losing more than the accepted success criteria of 5 and 10% of initial weight (*ps *< .001, for all comparisons). In addition, the body image and eating self-regulation variables included in the present model changed in the expected direction within the intervention group (*ps *< .001). Evaluative body image was enhanced, body image investment decreased, and eating self-regulation variables improved showing large effect sizes; significant between-group differences favoring the intervention were observed [55].

### Measurement Model

**Figure 1:**
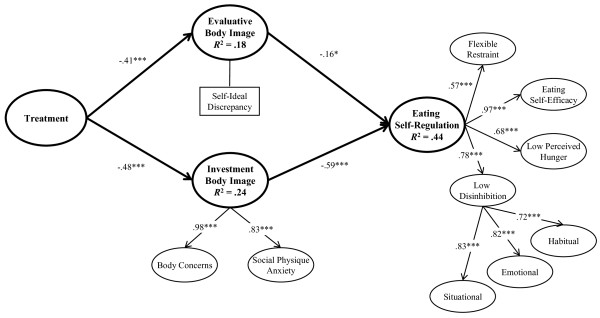
**Partial least squares model**. Values in the paths represent the bootstrapped PLS estimates; *p < .05, **p < .01, ***p < .001.

Initial PLS analysis showed that some observed indicators had low factor loadings (<.40) and some first-order latent variables presented AVEs below acceptable levels (.27 to .40). Therefore, the indicators with the lowest loadings were eliminated and the model re-estimated until acceptable AVEs were obtained. Figure 1 displays the lower-and higher-order LV's and the bootstrap estimates for the respective factor loadings. Table 1 shows the CRs, AVEs, and correlations among the latent variables. CRs for all scales were greater than .70 and AVEs .50 or larger. Moreover, AVEs for each latent variable were greater than the squared bivariate correlations with all the other latent variables, with the exception of the associations between lower-order variables and their respective higher-order LV, as expected. All correlations were significant (*p *< .05) and in the expected direction. Taken together, these findings suggest that the measurement model had acceptable internal consistency, convergent validity, and discriminant validity.

**Table 1:** Composite reliability (CR), average variance extracted (AVE) and correlations among factors in the measurement model

Correlations															
**Factor**	**CR**	**AVE**	**1**	**2**	**3**	**4**	**5**	**6**	**7**	**8**	**9**	**10**	**11**	**12**	**13**

1. Treatment (I vs C)	1	1	**1**												
2. *Investment BI*	.95	.91	-.48	**.95**											
3. Social Physique Anxiety	.87	.52	-.46	.83	**.72**										
4. Body Concerns	.95	.51	-.45	.98	.71	**.71**									
5. Evaluative BI	1	1	-.41	.36	.37	.32	**1**								
6. *Eating Self-Regulation*	.94	.75	.41	-.65	-.58	-.62	-.37	**.86**							
7. Flexible Restraint	.76	.51	.29	-.46	-.36	-.46	-.31	.57	**.71**						
8. Eating Self-Efficacy	.94	.53	.39	-.61	-.56	-.57	-.37	.97	.46	**.73**					
9. Perceived Hunger	.77	.53	.29	-.44	-.32	-.45^b^	-.18^a^	.68	.45	.54	**.73**				
10. *Disinhibition*	.83	.79	.33	-.57	-.51	-.55	-.26	.78	.39	.65	.57	**.89**			
11. Habitual Disin.	.80	.67	.23^b^	-.48	-.43	-.45	-.21^b^	.49	.36	.42	.35	.72	**.82**		
12. Emotional Disin.	.82	.60	.22^b^	-.40	-.36	-.38	-.20^a^	.59	.22	.49	.33	.82	.43	**.77**	
13. Situational Disin.	.79	.56	.33	-.51	-.45	-.49	-.22^b^	.75	.38	.62	.66	.83	.45	.49	**.75**

*Note*. N = 170. CR is composite reliability; AVE is average variance extracted; diagonal entries in bold are the square root of AVE; other values are correlation coefficients. Variables in italic are higher-order variables. ^a ^Correlations significant at *p *< .05; ^b ^Correlations significant at *p *< .01; All remaining correlations were significant at *p *< .001.

### Structural Model

The model explained between 18% and 44% of the variance in the dependent variables. Effect sizes were medium for the change in evaluative and investment body image (*f*^2 ^= .22 and .32, respectively), while large amounts of variance were explained for eating self-regulation (*f*^2 ^= .79). Figure 1 shows the PLS bootstrap estimates for the structural paths, and the variance accounted for in the dependent variables (*R*^2^).

Treatment positively predicted the change in body image investment and evaluative body dissatisfaction. Although both components improved significantly, treatment effects on the investment component were stronger (effect size .32 vs. .22). In turn, the positive changes in body image components resulted in an increase in eating self-regulation. Given the observed path coefficients, the effects of body image investment on eating self-regulation appear to be greater than the effects of evaluative body image (paths: -.59, p < .001 vs. -.16, p < .05). In the face of these results and to further support the greater relative strength of investment over evaluative body image effects on eating behavior, the model was re-examined before and after the inclusion of investment body image change. SmartPLS uses a blockwise estimation procedure, with only one part of the model being estimated at each time, which permitted the use of this additional analysis [48]. Results showed a substantial increase in variance explained in eating self-regulation (from an *R^2 ^*of .14 to .44) and a large effect size for change (*f^2 ^*= 0.54), further supporting a greater relative strength of investment over evaluative body image.

Table 2 shows the significant indirect effects between distal independent and dependent variables, and the resultant tests of mediation. Treatment had a significant indirect effect on eating self-regulation, which was fully mediated by the change in body image investment (effect ratio .68) and partially mediated by the change in evaluative body image (effect ratio .22). Results suggest that treatment effects on eating self-regulation occur especially through change in body image investment, given that the indirect effect via this dimension was greater than the one via evaluative body image (path coefficients: .28 vs .09).

**Table 2:** Significant indirect effects and tests of mediation in the structural model

	Relationship		Indirect effect ^a^ (ab path)	Total effect (C path)	Direct effect ^b^ (C' path)	Effect ratio
				
From	To	Intervening variable				
**Treatment**	**Eating self-regulation**	**Investment BI**	.28***	.41***	**.13**	**.68**
**Treatment**	**Eating self-regulation**	**Evaluative BI**	.09**	.41***	.32***	**.22**
						
Treatment	Flexible Restraint	Investment BI	.21***	.30***	.08	.70
Treatment	Eating self-efficacy	Investment BI	.27***	.39***	.13	.69
Treatment	Eating self-efficacy	Evaluative BI	.10**	.40***	.30***	.25
Treatment	Disinhibition	Investment BI	.26***	.33***	.06	.79
Treatment	Perceived hunger	Investment BI	.19***	.30***	.10	.63

*Note. N *= 170. BI: Body Image. All values represent the bootstrapped PLS estimates. ^a ^Whenever there is more than one intervening variable for each IV->DV path, the total indirect effect results from the sum of the indirect effect through each intervening variable. ^b ^Direct effect controlling for the mediator. *p < .05, **p < .01, ***p < .001.

To further explore the (mediating) role of body image change, secondary and more specific tests of mediation were conducted, considering each eating behavior as a separate outcome (see Table 2). Treatment had significant indirect effects on all measures of eating behavior (flexible restraint, eating self-efficacy, disinhibition, and perceived hunger). The change in investment body image fully mediated the effects of treatment on each one of these variables; the effect ratios were all large (.63 - .79). In addition, the positive change in body dissatisfaction partially mediated the path between treatment and eating self-efficacy (medium *f^2 ^*.25).

## Discussion

Body image problems are highly prevalent in overweight and obese people seeking treatment [56] and are consistently associated with poorer weight outcomes and increased chances of relapse [e.g., [6,8,11]]. In addition, poor body image has been consistently related to the adoption of maladaptive eating behaviors [e.g.,[16,17]], likely to undermine successful weight management. Thus, the advantage of tackling body image concerns in obesity treatment remains unquestioned. This study showed that body image improved during the intervention, confirming that behavioral weight loss programs, particularly those which include a body image module, can be an effective way of improving body image [25,57]. The present results extend previous findings by distinguishing evaluative and investment body image dimensions, showing that both can be enhanced, and that they differentially mediate the effects of a weight loss intervention on the (successful) regulation of eating behavior.

The conceptualized paths within the structural model were generally supported by the study's findings, accounting for a substantial portion of the variance in investment body image and eating-self-regulation. The study predictions were also generally supported. Specifically, results showed that the intervention led to positive changes in body image which in turn resulted in the improvement of eating self-regulation. In addition, results revealed that relative to evaluative body image, the change in body image investment was more strongly related to the changes in eating behavior. Finally, results showed that both body image dimensions mediated the significant effects of treatment on eating self-regulation. Overall, body image change appears to be a valid mechanism through which the regulation of eating behavior can be improved in behavioral weight management interventions, at least in women.

Results showed that this study's intervention led to improvements in both dimensions of body image, increasing body satisfaction, and decreasing dysfunctional investment in appearance. These findings lend support to previous suggestions by Rosen and colleagues [57,58] recommending the inclusion of body image-related contents in weight management interventions. Although we must acknowledge that some improvement in body image might have been experienced due to weight reduction per se, the rationale for adding a body image component to the intervention is that it will enable participants "to exercise their new self-image more effectively and to unlearn body image habits that do not give way to weight loss" [[59]; pp.436]. In addition, prior research suggested that body image enhancement could also facilitate the use of psychological resources, resulting in better adherence to the weight management tasks [60,61].

Change in both body image dimensions resulted in positive changes in eating self-regulation. Nevertheless, the present findings provide empirical support to the contention that reducing the levels of concern with body image (i.e., the investment in appearance) rather than body dissatisfaction is more strongly related to the successful adaptation of eating behavior. Besides the larger effect of investment change on eating regulation compared to the effect of evaluative body image, we observed a substantial increase in the variance explained in eating self-regulation (and a large *f^2 ^*for the change) after the inclusion of investment body image in the model. Previous research has shown that investment body image has more adverse consequences than evaluative body image to one's psychosocial functioning, and that dysfunctional investment in appearance is more associated with disturbed eating attitudes and behaviors than body dissatisfaction [21,23]. Explanation for these findings has been proposed to partially derive from a nuclear facet of body image investment, appearance-related self-schemas. These cognitive structures "reflect one's core, affect-laden assumptions or beliefs about the importance and influence of one's appearance in life, including the centrality of appearance to one's sense of self" [[62]; pp.42]. Appearance self-schemas derive from one's personal and social experiences and are activated by and used to process self-relevant events and cues [62,63]. According to Cash's cognitive-behavioral perspective [62], the resultant body image thoughts and emotions, in turn, prompt adjustive, self-regulatory actions (i.e., coping efforts), such as the adoption of dysfunctional eating behaviors [21,64]. In addition, Schwartz and Brownell [61] argued that body image distress could form a barrier to emotion regulation that, for both biological and psychological reasons, could result in increased (and unhealthy) eating. The present intervention significantly reduced participants' investment in appearance and its salience to their lives. Thus, it is possible that an increase in the acceptance of body image experiences and the deconstruction of held beliefs and interpretations about the importance of appearance to the self resulted in reduced appearance schemas' activation. In turn, this might have led to improvements in the regulation of associated thoughts and emotions, leading to the adoption of healthier and more adaptive self-regulatory activities [21].

In the present study, the effects of treatment on eating self-regulation were mediated by changes in both body image dimensions. To further explore these findings, more specific analyses of mediation were conducted considering each lower-order component of eating self-regulation as a separate outcome. Results suggested that the change in investment body image influenced all eating self-regulation variables, whereas the change in evaluative body image only mediated the improvement in eating self-efficacy. This finding could help explain why evaluative body image showed smaller effects in general; it mainly affected one of the four components of eating self-regulation used in this study. This finding is not surprising. Body dissatisfaction was assessed with a self-ideal discrepancy index which reflects change in current body size (through weight reduction) and/or change in ideal body size, for instance, by increasing acceptance of larger ideal body sizes [60,65]. In the face of more realistic and achievable ideal body sizes, individuals should feel more confident in making a compensatory aesthetic difference by losing some weight, namely via changes in eating behavior. In fact, prior research has suggested an association between seeing one's body as closer to the societal norm and self-efficacy for making healthy changes [c.f., [61]]. In addition, Valutis et al. [66] found that large body size discrepancies were related to disengaged coping efforts (i.e., reduced mental and behavioral energy put into change) due to low weight and eating-related self-efficacy. On the other hand, body image investment is related to the salience of appearance to one's life and sense of self [21] and is associated with negative affect [c.f., [17,62]] which makes it more likely to result in increased emotional eating, disinhibition and perceived hunger, and in the adoption of a rigid approach to eating.

The use of mediation analysis is a methodological strength of the present study. Mediation analysis is particularly well-suited to identify the possible mechanisms through which interventions achieve their effects, allowing the development of more parsimonious and effective interventions by emphasizing more important components and eliminating others [67]. Improving overweight and obesity interventions remains a critical challenge [68] and the present study represents one more step in this direction. This study was the first to explore body image as a mediator of eating self-regulation during weight control and to analyze the distinct effects of evaluative and investment body image components. The present findings are informative for professionals when designing future interventions, reinforcing the advantage of including a body image component within weight management treatments. Our results further suggest that within this intervention module, the strategies used to target body image investment should be emphasized to more effectively improve the regulation of eating behavior, and in turn more successfully manage body weight. This could be achieved by actively deconstructing and defying held beliefs and predefined concepts about the centrality of appearance to one's life and sense of self, mindfully accepting and neutralizing negative body image emotions, identifying problematic thoughts and self-defeating behavior patterns, and replacing them with healthier thoughts and behaviors [69]. This study was also the first to investigate eating self-regulation as a global, higher-order construct, represented by several variables previously identified as predictors of a successful eating/weight regulation (i.e., flexible cognitive restraint, eating self-efficacy, low disinhibition, and low perceived hunger) within overweight individuals [5,7]. Investigating specific mechanisms responsible for the successful regulation of eating behavior (e.g., increases in flexible cognitive restraint) is relevant as it will allow other weight loss interventions to focus on variables and components that are capable of effectively targeting behaviors already identified as predictors of successful weight management [5]. Future studies might find it important to continue to investigate this higher-order construct as a relevant outcome in weight loss interventions. This notwithstanding, the identification of other variables which may mediate the effects of treatment on eating self-regulation, for instance, related to physical activity [70], should be pursued.

Four limitations of the present study are noteworthy. First, although this was a longitudinal study and we did measure change in the variables of interest, changes in body image and eating measures occurred during the same period. Thus, we cannot exclude the possibility of alternative causal relations between these variables. It is possible that the change in eating self-regulation led to positive changes in body image, or that these variables reciprocally influence each other. However, based on the existing literature suggesting that poor body image is a precursor of dysfunctional eating behaviors [15,16,19], we hypothesized that it was the change in body image that resulted in positive changes in eating self-regulation. Second, the psychometric instruments used herein to measure investment body image were only able to capture some facets of this construct - over-preoccupation with body image and appearance and its behavioral consequences - thus failing to capture another core facet of body image investment, the appearance-related self-schemas. Future studies should include more comprehensive measures that are able to capture these additional facets of body image investment. Third, the format of the instrument used to assess evaluative body image has some inherent limitations. The Figure Rating Scale is a unidimensional and undifferentiated measure of body dissatisfaction that differs considerably from all other body image measures in format. By contrast, body image investment was assessed with more sophisticated and multidimensional instruments. This could account for the lesser role of the evaluative component in our model. Future studies should use multi-item questionnaire-type measures to assess evaluative body image. Finally, the generalizability of the findings in this study may be limited to overweight and obese women seeking treatment, a population that is particularly prone to body image disturbances, weight preoccupation, and dysfunctional eating patterns [7,56,71]. The effect of body image enhancement on eating self-regulation in other populations remains unknown.

## Conclusion

Results showed that both evaluative and investment body image are relevant for improving eating self-regulation during obesity treatment in women, and suggested that the investment component might be more critical. Professionals would do well to consider these findings when designing and implementing new interventions.

## Competing interests

The authors declare that they have no competing interests.

## Authors' contributions

EVC, PJT, and DM conceived the study. EVC performed the statistical analysis, participated in the intervention and data collection, and drafted the manuscript. MNS led the implementation team and actively participated in the intervention's implementation and data collection. PNV and CSM actively participated in the intervention's implementation and in data collection. DM provided additional statistical advisement. PJT is a principal investigator of the trial and participated in drafting the final version of the manuscript. LBS is a principal investigator in the research trial. All authors read and approved the final manuscript.
